# MOF-Based Mycotoxin Nanosensors for Food Quality and Safety Assessment through Electrochemical and Optical Methods

**DOI:** 10.3390/molecules27217511

**Published:** 2022-11-03

**Authors:** Hessamaddin Sohrabi, Parya Salahshour Sani, Ramin Zolfaghari, Mir Reza Majidi, Yeojoon Yoon, Alireza Khataee

**Affiliations:** 1 Department of Analytical Chemistry, Faculty of Chemistry, University of Tabriz, Tabriz 51666-16471, Iran; 2 Department of Inorganic Chemistry, Faculty of Chemistry, University of Tabriz, Tabriz 51666-16471, Iran; 3 Mining Engineering Faculty, Amirkabir University of Technology, Tehran 15875-4413, Iran; 4 Department of Environmental and Energy Engineering, Yonsei University, Wonju 03722, Korea; 5 Research Laboratory of Advanced Water and Wastewater Treatment Processes, Department of Applied Chemistry, Faculty of Chemistry, University of Tabriz, Tabriz 51666-16471, Iran; 6 Department of Environmental Engineering, Gebze Technical University, 41400 Gebze, Turkey; 7 Department of Material Science and Physical Chemistry of Materials, South Ural State University, 454080 Chelyabinsk, Russia

**Keywords:** MOF-based compounds, mycotoxins, food quality, electrochemical and optical methods

## Abstract

Mycotoxins in food are hazardous for animal and human health, resulting in food waste and exacerbating the critical global food security situation. In addition, they affect commerce, particularly the incomes of rural farmers. The grave consequences of these contaminants require a comprehensive strategy for their elimination to preserve consumer safety and regulatory compliance. Therefore, developing a policy framework and control strategy for these contaminants is essential to improve food safety. In this context, sensing approaches based on metal-organic frameworks (MOF) offer a unique tool for the quick and effective detection of pathogenic microorganisms, heavy metals, prohibited food additives, persistent organic pollutants (POPs), toxins, veterinary medications, and pesticide residues. This review focuses on the rapid screening of MOF-based sensors to examine food safety by describing the main features and characteristics of MOF-based nanocomposites. In addition, the main prospects of MOF-based sensors are highlighted in this paper. MOF-based sensing approaches can be advantageous for assessing food safety owing to their mobility, affordability, dependability, sensitivity, and stability. We believe this report will assist readers in comprehending the impacts of food jeopardy exposure, the implications on health, and the usage of metal-organic frameworks for detecting and sensing nourishment risks.

## 1. Introduction

Steady population development and business advancements make food security issues severe in the ecosystem, with serious public health implications. Nutrition security has an indirect impact on various financial, social, and ecological interactions involving food production, resulting in environmental consequences for farming, food trading, and energy consumption [1,2]. Foodborne diseases are a global issue, and food quality evaluation is significant to human health, determining whether food can be distributed and used in the marketplace. The presence of food contaminants, including veterinary drugs, pesticide residues, toxins, illegal artificial additives, heavy metal ions, pathogens, and mycotoxins, has raised concerns about food safety [3,4]. Contaminants that are usually problematic are marine biotoxins, mycotoxins, harmful toxins occurring in poisonous mushrooms, and cyanogenic glycosides [5]. The mycotoxin rubric was broadened to embrace previously documented fungal toxins (e.g., ergot alkaloids, trichothecenes), compounds first identified as antibiotics, such as patulin (PAT), citrinin (CIT), and new secondary metabolites discovered in mycotoxin discovery screens, such as ochratoxin A (OTA) and zearalenone [6,7]. Saprophytic molds and filamentous fungi, such as *Fusarium*, *Aspergillus*, and *Paecilomyces*, are the best-known poisonous mushrooms whose second metabolites produce a toxin called mycotoxins [8]. It has been demonstrated that pathogenic fungi, particularly *Fusarium* species, can continue to generate mycotoxins in water, providing a potential pathway for human exposure to mycotoxins through surface water [9]. Additionally, humans can be poisoned by food and feed; mycotoxins can contaminate food or food crops at all stages of the food chain [10]. The presence of mycotoxins has been related to the development of a variety of illnesses that disturb the regular functioning of healthy cells in both humans and animals. Cancer, renal toxicity, birth abnormalities, immunological suppression, autism, bleeding, neurotoxicity, chronic fatigue syndrome, memory loss, asthma, depression, acute pulmonary, anemia, and sinusitis are among the clinical signs and severe health consequences associated with their exposure [11,12]. Therefore, identifying mycotoxins in diverse foods and beverages through real-time and on-site testing is essential to ensure safety. The sensing of mycotoxins needs to be distinguished from the detection of fungi that produce mycotoxins [13].

Mycotoxins are traditionally detected using chromatographic methods, such as high-performance liquid chromatography (HPLC) with fluorescence detection [14], HPLC with ultraviolet (UV) detection [15], thin-layer chromatography (TLC) with the ability to analyze images or quantify densitometry [16], gas chromatography with various detectors, and chromatographic separation combined with mass spectrometry [17]. In addition to chromatographic methods, plasma mass spectrometry is inductively linked to gas chromatography-mass spectrometry (GC-MS) [18,19], atomic absorption spectrometry (AAS) [20], and ion mobility spectrometry (IMS) [21]. Although these techniques have many desirable properties and can meet many conditions required in this field, their application in routine food monitoring is limited by laborious sample preparation and specialized devices. Consequently, sensing technology has been significantly considered in recent years [22]. Sensing assays are small analytical systems with bio-recognition and transmission sensors that allow for the precise detection of various analytes and convert analyte identification into physically quantifiable optical and electrical signals [23,24]. Electrochemical biosensors are typically developed based on proximity-dependent interactions mediated electrically between labeled probes and nanomaterial-coated electrodes [25,26]. These sensors are rapid, easy to handle, sensitive, and simple to use, matched with cost-effective instruments without sample preparation. A category of biosensors with rapid detection, straightforward procedure, pale operation, high sensitivity, sizeable linear range, and cost efficiency is optical biosensors with a detecting device that interacts visually with the target analyte and a signaling-related transducer component [27,28]. They are frequently used in analytical and experimental testing for food safety assessment processes and monitoring food packaging to develop efficient new strategies [28,29].

Recently, metal-organic framework (MOF)-based sensors for nutrition safety management, notably for detecting mycotoxins, have been developed because of their accuracy, sensitivity, rapidity, and ease of use for online monitoring and control of dangerous compounds, such as mycotoxins in different food samples [30,31]. Furthermore, as a new class of porous crystalline materials, MOFs have the advantages of homogeneous structures, tunable composition, ultra-high porosity, simple functionalization on their surface interfaces, and optoelectronic capabilities [32,33]. Therefore, the primary purpose of this review is to present the most recent and comprehensive overview of the current status of MOF-based electrochemical and optical sensing assays for detecting mycotoxins in food products.

## 2. MOFs: Types, Various Synthesis Methods, and Applications

MOFs are crystalline porous polymers composed of coordination bonds. Metal ions and organic linkers are commonly used to fabricate MOFs via a solvothermal process that occurs at a specific temperature and time. Various synthetic approaches, including microwave-assisted, electrochemical, mechanochemical, sonochemical, and layer-by-layer growth syntheses, have been described in addition to solvothermal synthesis [34]. MOFs exhibit remarkable adsorption characteristics owing to their distinctive physicochemical characteristics and persistent interior porosity. They are also efficiently designed and synthesized by grafting several groups (such as -COOH and -NH_2_) either in situ or after they have been synthesized or purified [35,36]. These characteristics make MOFs excellent candidates for co-immobilizing biological ligands via strong interactions, such as stacking, hydrogen bonding, and electrostatic forces between the functional groups of the MOF and the biological ligands, which have the potential to be helpful in the development of biosensors [37]. Recently, there has been much interest in applying MOFs to fabricate sensors to check food safety. This includes investigations on luminescence [38], electrochemical [39], colorimetric [40], and surface-enhanced Raman scattering (SERS) sensors [41]. Although these sensors employ various detection approaches, their sensing performance is promising for food safety monitoring [42,43].

Typically, MOFs are produced using sol-gel and various other methods, such as (a) electrochemical technique (continuous and fast microcrystalline MOF production) [44]; (b) hydro/solvothermal technique ([high-quality MOF crystals, many days of reaction temperature, pressure > 100 °C]; some common solvents in this method are alcohols, acetonitrile, acetone, dimethyl sulfoxide (DMSO), N,N-dimethylformamide (DMF), N,N-dimethylformamide (DEF) and H_2_O) [45]; (c) slow solvent evaporation technique (traditional method generated under ambient conditions, but with a very long reaction time) [46]; (d) microwave-assisted heating technique (brief nucleation time and uniform pore shapes and sizes) [47]; (e) mechatronics technique (ecological, economic, short reaction time, no formation of polluting or toxic compounds, and solvent-free) [48]; and (f) sonochemical reaction technique (homogeneous nucleation by cavitation revealed by ultrasound) [49]. All these procedures are equally sensitive to changes in the type of solvent, reagent concentration, molar ratio of the starting materials, reaction pH, counter ions, pressure, temperature, and timeframe [50].

## 3. Electrochemical Platforms for Sensing Different Mycotoxins in Various Foods

Electrochemical sensing assays are self-contained, integrated analytical systems in which a biological sensing element is close to or integrated with an electrochemical transmitter, allowing the analytical response to be measured using various electrochemical methodologies, such as amperometric, potentiometric, impedimetric, conductometric, or field effects [51]. The surfaces of electrochemical sensors can contain surfactants, metals, inorganic or organic molecules, biomaterials, MOF-electroactive compounds, nanoparticles (NPs), and nanocomposites [52]. By integrating the cognitive and transformational aspects, electrochemical sensors can recognize qualitative and quantitative analytics and transform raw electrochemical data into analytical signals. They require no sample preparation and involve inexpensive equipment, simple operation, rapid processing, and better sensitivity than conventional techniques [53]. Electrochemical reactions occur between or on the electrode surfaces, restoring the redox equilibrium between the electrolyte and the molecule or ion of interest. Scientists have investigated multiple materials as potential sensor substrates [54]. In particular, MOFs are distinguished by their porosity, morphologies, pore diameter, high specific surface area, electro-optical properties, and multimodal behaviors, which assist enhanced electrochemical sensors [55,56]. Consequently, the use of MOFs with electrochemical activity appropriate for changing electrodes to monitor a range of pollutants, particularly mycotoxins from supply sources, food packaging, food preservation, and detection and monitoring of various food products, has been enhanced [57].

**Figure 1:**
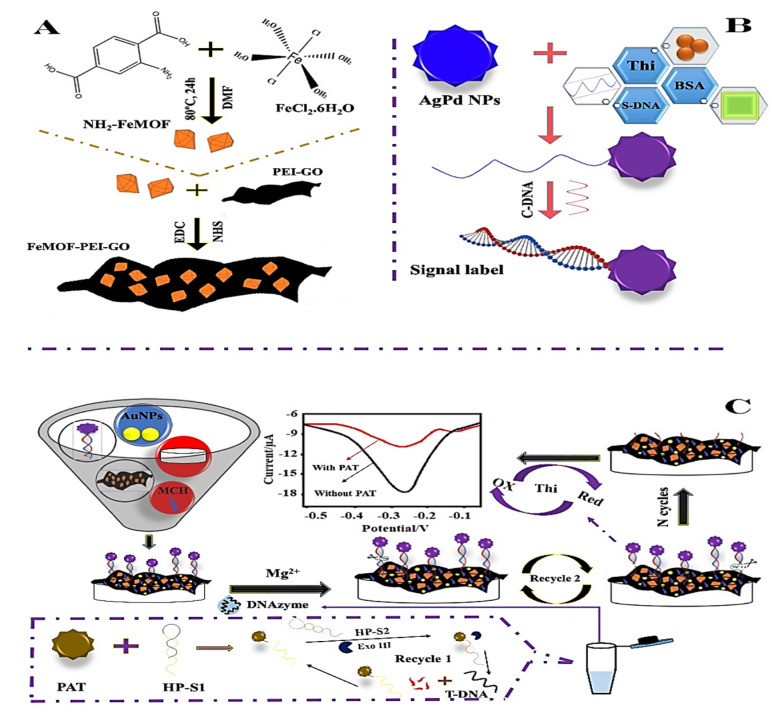
(**A**) Graphical representation of FeMOF-PEI-GO and NH_2_-FeMOF synthesis: (**B**) Nanoprobe fabrication; (**C**) Schematic representation of electrochemical aptasensor and patulin (PAT) detection. Adapted with permission from the article of [58]. 2022, Elsevier.

Numerous efforts have been made to examine the detection and elimination capabilities of electrochemical sensing (Table 1). In this context, Lu et al. (2022) [58] proposed an AuNPs/FeMOF-PEI-GO (Figure 1A,B) aptasensor with a detection range of 5 × 10^−10^ mg/L to 0.005 mg/L and a low detection limit (LOD) of 2.17 × 10^−10^ mg/L to sense patulin (PAT) in apple juice with reliability, validity, and extended stability, offering a valuable method for analyzing trace patulin precisely in the area of food safety field (Figure 1C).

**Figure 2:**
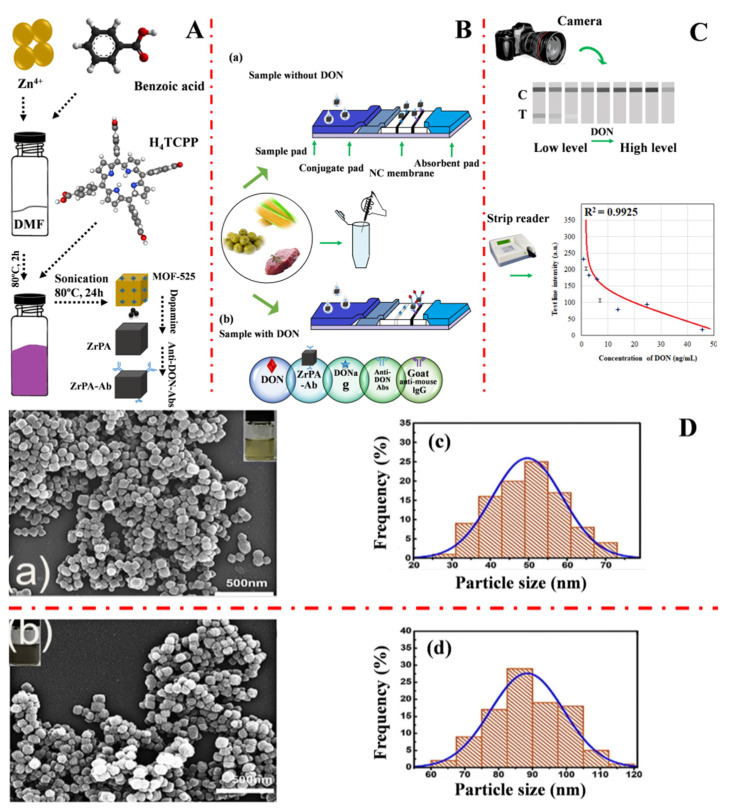
(**A**) Graphical illustration of the MOF-525, ZrPA, and ZrPA-Ab synthesis stages; (**B**) The ZrPA-working ICA concept of sensing deoxynivalenol; (**C**) Interpretation of the findings of the experiment, and (**D**) (**a**,**b**) MOF-525 and (**c**,**d**) ZrPA SEM images and particle sizes, respectively. Adapted with permission from the article of [59]. 2022, Elsevier.

Rui Li et al. (2020) [59] developed a conventional immunochromatographic assay biosensor capable of detecting deoxynivalenol by employing zirconium metal-organic frameworks labeled antibodies (ZrPA-Ab) as a probe and using a simple oxidative self-polymerization assembly (OPMA) technique. Figure 2A shows a graphical illustration of the synthesis of MOF-525, ZrPA, and ZrPA-Ab. The ZrPA-working ICA concept for sensing deoxynivalenol is shown in Figure 2B. The interpretation of the results is explained in Figure 2C. The morphological information of MOF-525 and ZrPA SEM images and the particle sizes are shown in Figure 2D(c,d). ZrPA-ICA had detection limits of 0.00018 mg/L and a range of 0 to 0.05 mg/L in green bean, millet, maize, and pork hind legs.

**Figure 3:**
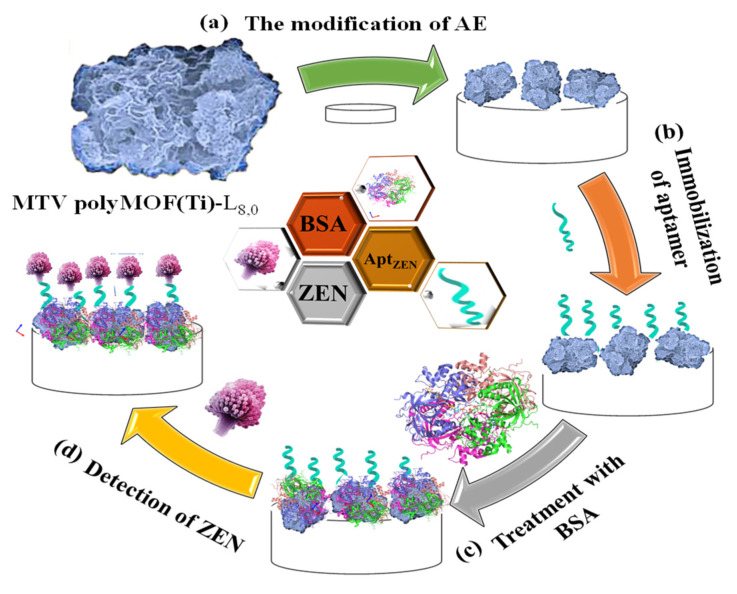
Graphical illustration of the stages involved in creating an aptasensor relying on MTV poly MOF-L8,0 for the sensing of zearalenone, comprising modifying the Au electrode with MTV poly MOF-L8,0, zearalenone-targeted aptamer anchoring, Apt/MTV poly MOF-L8,0/AE blocking, and zearalenone sensing utilizing bovine serum albumin (BSA)/Apt/MTV poly MOF-L8,0/AE. Adapted with permission from the article of [64]. 2022, Elsevier.

**Figure 4:**
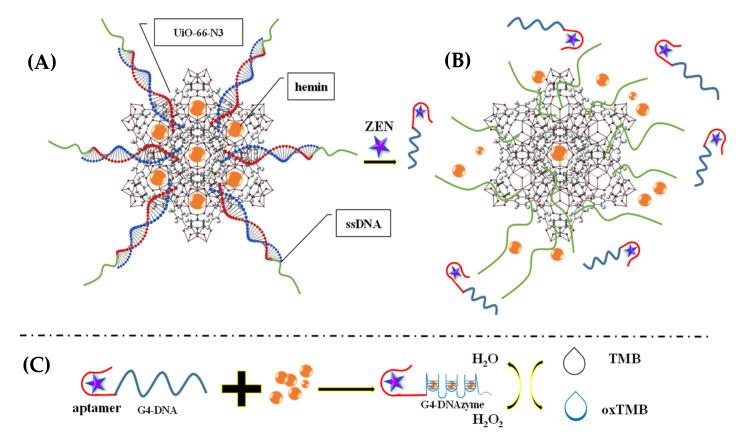
(**A**) Hemin-entrapped MOF synthesis gated by a duplex cDNA/ssDNA, where the ssDNA contains the trimeric G4-DNA sequence and the zearalenone aptamer sequence. (**B**) The following section describes how the zearalenone/aptamer complex releases the cDNA/ssDNA-gated, hemin-entrapped MOF. (**C**) Graphical depiction of the production of G4-DNAzyme and its catalytic function. Adapted with permission from the article of [65]. 2022, Elsevier.

Moreover, Gurjeet Kaur et al. (2022) [60] developed a MoS_2_ QDs@UiO-66-NH2 sensor by introducing screen-printed carbon electrodes with a functional nanohybrid comprising a quantum dot (QDs) zirconium-based MOF and MoS_2_, namely, UiO-66-NH_2_. The modified MoS_2_/UiO-66 electrodes were coated with monoclonal antibodies specific to aflatoxin M1 (AFM1) and subsequently evaluated for electrochemical sensing of AFM1. Electrochemical impedance spectroscopy analysis identified AFM1 at concentrations between 0.21 mg/L and 6.0 × 10^−5^ mg/L with a detection limit of 6.0 × 10^−5^ mg/L. Various fabrication steps are extensively demonstrated. In addition, to detect aflatoxin B1 (AFB1) in a sample of rice flour, Fahime Jahangiri–Dehaghani et al. (2022) [61] developed AuNPs decorated on a Ni-MOF nanosheet (AuNP/Ni-MOF) with a linear range of 5.0 × 10^−6^–0.15 mg/L and 1.0 × 10^−6^ mg/L limit of detection. In 2021, Fahime Jahangiri–Dehaghani et al. developed a non-label electrochemical aptasensor based on the Cu MOF to detect AFB1 in wheat flour samples. This aptasensor had a linear range of 1.0 × 10^−6^ mg/L to 0.2 mg/L and an 8.3 × 10^−7^ mg/L limit of detection [62]. In addition, Zhou Xu et al. (2021) [63] developed an indirect competitive MOF enzyme-linked immunosorbent assay (MOFLISA) to identify AFB1 in soy milk and nested peanut milk samples. Using MOFLISA technology, they demonstrated substituting natural enzymes, such as horseradish peroxidase (HRP) with antibodies bound to MIL-88. This biosensor has a lower detection limit (9.0 × 10^−6^ mg/L) and is more stable than the usual ELISA, which has a linear operating range of 1.0 × 10^−5^–0.02 mg/L. In another study, [pbdc-xa or L8, pbdc = poly (1,4-benzene dicarboxylate) and 1,4-benzene dicarboxylic acid (H2bdc or L0] MTV poly MOF(Ti)-L8,0 was developed by Duan et al. (2022) [64] based on a multivariate titanium metal-organic framework, that is, MTV poly MOF, to sense zearalenone in peanut beer and corn (Figure 3). This framework had a detection limit of 7 × 10^−9^ mg/L level in electrochemical impedance spectroscopy (EIS) and 3.5 × 10^−9^ mg/L in the differential pulse voltammetry (DPV) technique within the 10 × 10^−9^ mg/L to 0.01 mg/L linear range. Similarly, in 2022, Yuhan Sun et al. [65] reported a colorimetric aptamer-functionalized MOF-nanocontainer and a trivalent DNA peroxidase mimicking enzyme (DNAzyme) based on a cDNA/ssDNA stimuli-responsive with the linear range of 0.00001–0.1 mg/L and a detection limit of 3.6 × 10^−7^ mg/L to detect zearalenone in maize and wheat samples. Figure 4A shows hemin-entrapped MOF synthesis gated by a duplex cDNA/ssDNA, where the ssDNA contains the trimeric G4-DNA sequence and the zearalenone aptamer sequence. The following section describes how the zearalenone/aptamer complex releases the cDNA/ssDNA-gated, hemin-entrapped MOF (Figure 4B), and a graphical depiction of the production of G4-DNAzyme and its catalytic function is shown in Figure 4C.

Zeng et al. (2022) [66] developed vast surface area, strong electron transport, and electrochemical nanohybrid sensors by employing a layer-by-layer assembly method. These sensors were based on magnetic Fe_3_O_4_-graphene oxide (Fe_3_O_4_-GO)-modified electrodes and (Cu-MOF). As a result, zearalenone was identified in rice flour, maize powder, and breakfast cereal in a linear range of 0.1592–2.8652 mg/L, with a detection limit of 0.023 mg/L.

**Figure 5:**
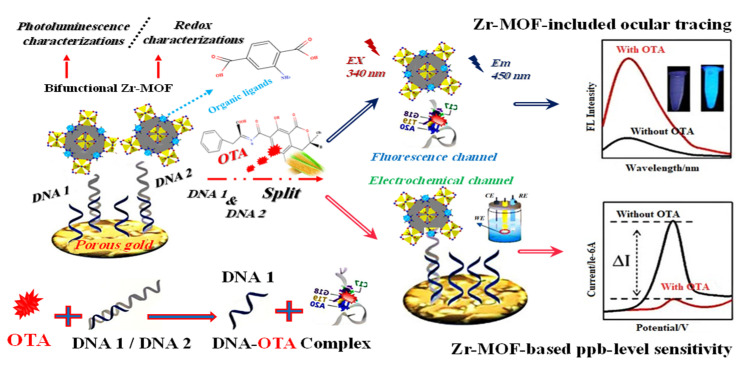
Graphical description of the dual-channel detecting of ochratoxin A (OTA). Adapted with permission from the article [67]. 2022, American Chemical Society.

Additionally, Lin et al. (2022) [67] demonstrated a Zr-MOF highly porous gold electrochemical and fluorescence dual-channel biosensor with a 1.0 × 10^−7^–0.14 × 10^−3^ mg/L linear range and 2.4 × 10^−8^ mg/L detection limit sensing ochratoxin A (OTA) in corn samples. Additionally, this sensor showed fluorescence intensity positively connected with that of OTA throughout a concentration range of 1.0 × 10^−7^ to 0.16 × 10^−3^ mg/L, with a detection limit of 5.1 × 10^−8^ mg/L. In Figure 5, DNA1 was put on a sensing substrate made of highly porous gold (HPG) to boost electrochemical signals. Because DNA2 recognized OTA in a very specific way, the anchored Zr-MOF probe was released from the sensing interface and into the reaction solution. This made the electrochemical signal weaker and the fluorescence response stronger.

**Figure 6:**
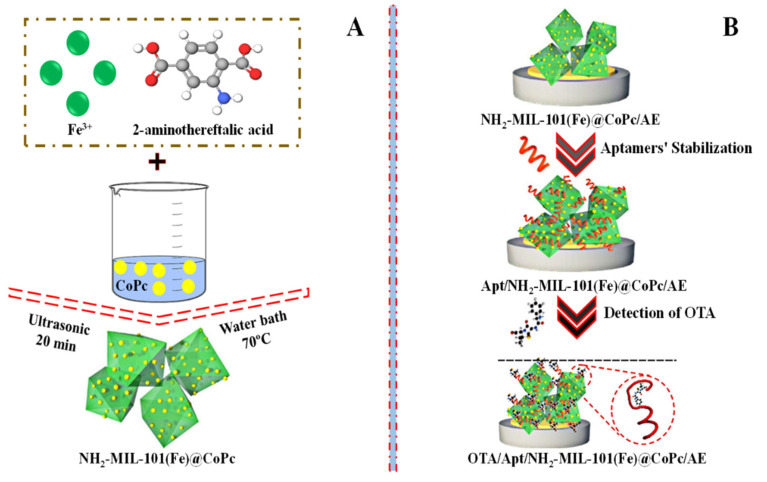
(**A**) Graphical depiction of the synthesis of NH_2_-MIL-101@CoPc nanocomposite and (**B**) the construction of an aptasensor based on NH_2_-MIL-101@CoPc for the detection of OTA. Adapted with permission from the article [68]. 2022, Elsevier.

In addition, to detect OTA, an iron-based metal-organic structure (NH_2_-MIL-101) was mixed with various doses of cobalt phthalocyanine nanoparticles (CoPc) to prepare a range of NH_2_-MIL-101@CoPc nanocomposites (Figure 6A). The aptasensor based on (NH_2_/MIL-101(Fe)@CoPc 6:1) showed a performance for sensing OTA in watermelon and wine with a low detection limit (LOD) of 0.063 × 10^–9^ mg/L and 1.0 × 10^−10^ to 1.0 × 10^−4^ mg/L linearity range [68]. The construction of a sensing assay based on NH_2_-MIL-101@CoPc to detect OTA is schematically shown in Figure 6B.

Zhang et al. (2020) [69] established an AgPt/PCN-223-Fe electrochemical tracer with AgPt bimetallic nanoparticles decorated with an iron-porphyrinic metal-organic structure (PCN-223-Fe) to sense OTA. The suggested sensor demonstrated a low detection limit of 14 × 10^−9^ mg/L (S/N = 3) and a linear range of 20 × 10^−9^ mg/L to 0.002 mg/L in red wine and corn, respectively.

**Figure 7:**
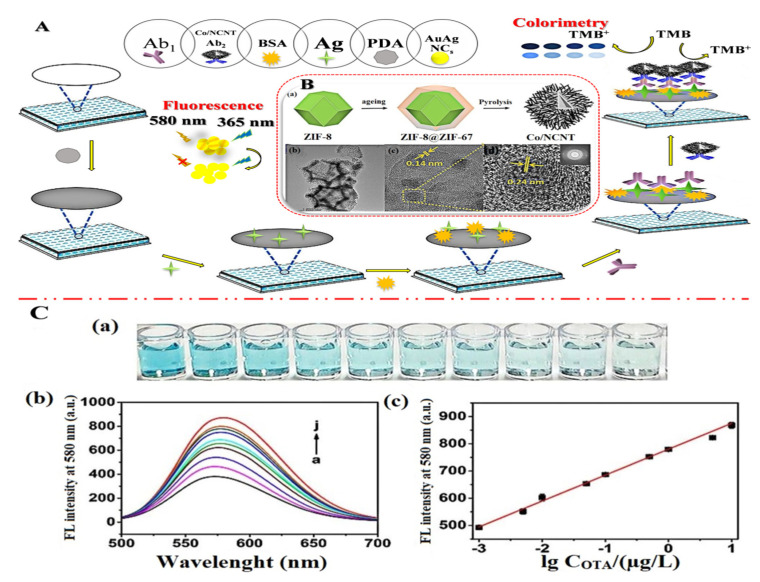
(**A**) Diagrammatic representation of a dual-mode immunosensor that mimics oxidase utilizing Co/NCNT: (**B**) (**a**) The Co/NCNT synthesis method; the low-resolution (**b**) and (**c**,**d**) high-resolution TEM pictures, as well as the matching electron diffraction images (illustration of **d**), EDS; (**C**) (**a**) colorimetric photos, (**b**) fluorescence spectroscopy, and (**c**) ochratoxin A (OTA) calibration plot with various concentrations (a–j: 0, 0.001, 0.005, 0.01, 0.05, 0.1, 0.5, 1, 5, 10 μg/L). Adapted with permission from the article [70]. 2022, Elsevier.

**Figure 8:**
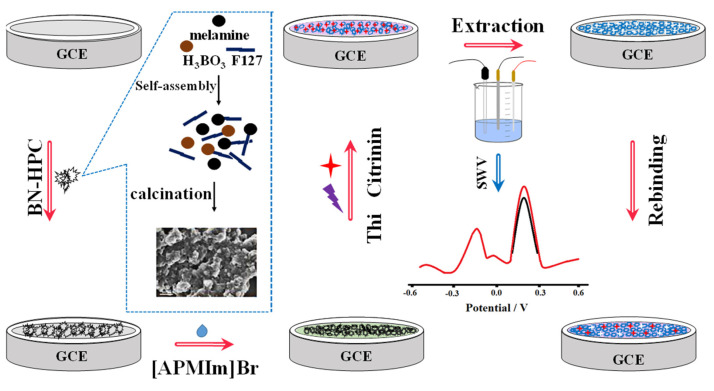
Schematic representation for the manufacture and operation of MIP/[APMIm]Br/BN-HPC/GCE, as well as the preparation process for BN-HPC (with permission from the article [71]).

In addition, by high-temperature carbonization of the precursor ZIF-8@ZIF-67, Mengting Chen et al. (2022) [70] developed a colorimetric-fluorescent immune sensor for the Co nanoparticle/N-doped carbon nanotubes (Co-NCNT) with a hollow core-shell on ZIF-8@ZIF-67. This structure not only served as a source of carbon and nitrogen for NCNTs catalyzed by cobalt nanoparticles generated in situ, but it also served as a template for developing the hollow structures applied to sense OTA concentration in commercial millet and corn samples with 10 × 10^−6^ to 0.01 mg/L linearity detection range and low detection limit of 2.1 × 10^−7^ mg/L and 1.7 × 10^−7^ mg/L for colorimetric and fluorescence techniques, respectively. Figure 7A shows the dual-mode immunosensor that mimics oxidase using Co/NCNTs. Moreover, Figure 7B shows the Co/NCNT synthesis method. TEM images are shown in Figure 7B(a–d) to investigate the morphological characteristics. Figure 7C(a,b) shows the colorimetric instigations and fluorescence spectroscopy, respectively. Finally, a calibration plot with various concentrations is depicted in Figure 7C(c). Xiaopeng Hu et al. (2021) [71] announced the construction of a molecularly imprinted polymer-based ratiometric electrochemical sensor [MIP/(boron and nitrogen co-doped hierarchical porous carbon) BN-HPC/1-aminopropyl-3-methylimidazolium bromide ([APMIm]Br)/GCE] to detect citrinin in rice, wheat, and red yeast rice samples. This sensor exhibited a linearity range of 1.0 × 10^−6^ to 0.01 mg/L and a detection limit of 1.0 × 10^−7^ mg/L. Figure 8 depicts poly (Thi) as a reference, and [Fe(CN)_6_]^3−/4−^ acted as indicating probes; then, ionic liquid (i.e., 1-aminopropyl-3-methylimidazolium bromide, [APMIm]Br) was immobilized on the surface of boron and nitrogen co-doped hierarchical porous carbon (BN-HPC) to increase the reliability of the MIP-RECS in detecting citrinin.

In another research project, Xiaoyan Wen et al. (2021) [72] modified an electrochemical aptasensor based on Cu–N–MOF. This sensor had a 2.0 × 10^−5^ to 0.02 mg/L (R^2^ = 0.994) linearity concentration range with an 8.0 × 10^−6^ mg/L detection limit to detect deoxynivalenol, one of the most prominent mycotoxins in the contaminated wheat sample.

**Table 1:** Recent advances in electrochemical-based sensing platforms for detecting various kinds of mycotoxins in numerous food samples.

MOF Type	Technique	Target Mycotoxins	Modifier	Detection Limit (LOD)	Linear Range	Recovery (%)	Reference
AFB1-PBP-cDNA-Apt-MPA-AuNP-NiMOF-GCE	DPV	Aflatoxin B1 (AFB1)	AuNP/Ni-MOF	1.0 × 10^−6^ mg·L^−1^	5.0 × 10^−6^–0.15 mg·L^−1^	8.4–101.3	[61]
AuNPs/FeMOF-PEIGO; AgPdNPs	DPV	Patulin (PAT)	AuNPs/FeMOF-PEI-GO	2.17 × 10^−10^ mg·L^−1^	5 × 10^−10^–0.005 mg·L^−1^	91.0–103	[58]
MTV polyMOF-L_8,0_	DPV and EIS	Zearalenone	ligand of L8 or L0	7 × 10^−9^ mg·L^−1^ and 3.5 × 10^−9^ mg·L^−1^	10 × 10^−9^ mg L^−1^ to 0.01 mg·L^−1^	95.72–106.32	[64]
MoS_2_ QDs@UiO-66-NH_2_ composite	CV and EIS	Aflatoxin M1 (AFM1)	UiO-66-NH_2_	6.0 × 10^−5^ mg·L^−1^	0.0002−0.01 mg·L^−1^	-	[60]
N–Cu–MOF	DPV	Deoxynivalenol [16]	N-Doped	8.0 × 10^−6^ mg·L^−1^	2.0 × 10^−5^–0.02 mg·L^−1^	95.6–105.9	[72]
Zr-MOF	EIS	Ochratoxin A (OTA)	-	2.4 × 10^−8^ mg·L^−1^	1.0 × 10^−7^–0.14 × 10^−3^ mg·L^−1^	-	[67]
CuMOF-GCE	EIS	Aflatoxin B1 (AFB1)	CuMOF	8.3 × 10^−7^ mg·L^−1^	1.0 × 10^−7^–0.2 mg·L^−1^	97.8–105.5	[62]
NH_2_/MIL-101@CoPc_6:1_	EIS and CV	Ochratoxin A (OTA)	CoPc_6:1_	0.063 × 10^−9^ mg·L^−1^	1.0 × 10^−10^–1.0 × 10^−4^ mg·L^−1^	98.2–110.0	[68]
Cu-MOF/Fe_3_O_4_-GO	DPASV	Zearalenone (ZEA)	Cu-MOF	0.023 14 mg·L^−1^	0.1592–2.8652 mg·L^−1^	96.4–97.3	[66]
MOFLISA (MOFs@Ab_2_)	chromogenic system	Aflatoxin B1 (AFB1)	ELISA	9.0 × 10^−6^ mg·L^−1^	1.0 × 10^−5^ to 0.02 mg·L^−1^	86.41–99.77	[63]
MIP/BN-HPC/[APMIm]Br/GCE	SWV	Citrinin	[APMIm]Br/BN	1.0 × 10^−7^ mg·L^−1^	1.0 × 10^−6^−0.01 mg·L^−1^	97–110	[71]
SA/AgPt/PCN-223-Fe	DPV	Ochratoxin A (OTA)	AgPt	14 × 10^−9^ mg·L^−1^	20 × 10^−9^–0.002 mg·L^−1^	95.5–104.0	[69]
ZrPA-ICA	Immunochromatographic	Deoxynivalenol [16]	ZrPA	0.18 × 10^−3^ mg·L^−1^	0.18 × 10^−3^–0.05 mg·L^−1^	97.8–109.5	[59]
Cu-MOF/AuNPs/S4	DPV	Aflatoxin B1 (AFB1)	DNA (S4)	6.7 × 10^−10^ mg·L^−1^	1.0 × 10^−9^–0.001 mg·L^−1^	96–103	[73]
SA/Au NPs@Cd/MOF-74	DPV	Ochratoxin A (OTA)	Cd-MOF-74	1.0 × 10^−5^ mg·L^−1^	5.0 × 10^−5^–0.1 mg·L^−1^	91.1–105.2	[74]
BSA/Apt-PtNP/MIL–101/GCE	EIS	Aflatoxin M1 (AFM1)	MIL–101	2.0 × 10^–6^ mg·L^−1^	1.0 × 10^–5^–0.08 mg·L^−1^	93.0–108.0	[75]
AuNP/MIP-MOF	LSV	Aflatoxin B1 (AFB1)	AuNP	0.3 × 10^−9^ mg·L^−1^	0.0000032 nM–3200 nM	-	[76]
AuE/DLS/OBA-TSS/UiO-66/MCH	SWV	Ochratoxin A (OTA)	UiO-66	7.9 × 10^−8^ nM	10^−7^–2000 nM	98.5–103.7	[77]
Zr-MOFs-PEI-rGO/Fe-MOFs/Pt@AuNRs	DPV	Patulin (PAT)	MB@Zr-MOFs-cDNA	4.14 × 10^−8^ mg·L^−1^	5.0 × 10^−8^–0.0005 mg·L^−1^	87–101	[78]
DNA-PtNi@Co-MOF/AuNRs/CoSe2	DPV	Zearalenone [44]	PtNi@Co-MOF	1.37 × 10^−9^ mg·L^−1^	10×10^−9^–0.01 mg·L^−1^	93.6–103.4	[79]
MIP-Au@Cu-MOF/N-GQDs/GCE	DPV	Patulin (PAT)	Au@Cu-MOF	7.0 × 10^−7^ mg·L^−1^	1.0 × 10^−6^–0.07 mg·L^−1^	97.6–99.4	[80]
MIP-Au@PANI-SeS_2_@Co MO	DPV	Patulin (PAT)	SeS_2_@Co MO	0.001–100 nM	0.001–100 nM	94.5–106.4	[39]
CoNi-MOF	EIS	Deoxynivalenol [16]	CoNi	5.0 × 10^−8^ mg·L^−1^	1.0 × 10^−6^–0.0005 mg·L^−1^	95.7–102.6	[81]
Ag NPs/2D MOF sheets	DPV	Ochratoxin A (OTA)	Ag NPs	0.08 × 10^−9^ mg·L^−1^	0.10 × 10^−9^–1 mg·L^−1^	99.27–101.20	[82]
MIP/COFs-AuNPs/AuE	ELISA	Aflatoxin B1 (AFB1)	COFs-AuNPs	2.8 × 10^−6^ mg·L^−1^	5.0 × 10^−5^–0.075 mg·L^−1^	87.0−101.7	[83]

## 4. Optical Sensing Platforms for Sensing Different Mycotoxins in Various Food

Fluorescent, chemiluminescent (CL), and electrochemiluminescence (ECL) optical sensors depend on various luminescent mechanisms. The fluorescent functional MOF composites display unique chemical characteristics. Once guest molecules or ions arrive at the MOF pores, they experience various levels of fluorescence intensification or quelling reactions [84]. Owing to its high sensitivity, broad linear limit, and needless stimulus light sensor, a chemiluminescence sensor is a potent gadget for major chemical analyses. The application of MOF-based composites can enhance the luminescence yield of CL systems [85]. Optical sensing assays can use optical fibers, planar waveguides, SPR, and microarrays. They detect the light amplitude, phase, frequency, or polarization. They have several improvements over the older methods. The use of MOF-based biosensors is rapidly increasing [86]. MOFs’ chemical and physical features provide optical MOFs light, and their topologies define MOF-optical sensors. This depends on the complexity of the MOFs’ building ingredients. The fluorescence might originate through metal centers and ligands, and optical qualities can be adjusted by modifying structural intricacy. In addition, photoresponsive components can be added to MOFs to induce fluorescence for various purposes [87,88]. Luminescent MOFs (LMOFs) respond to changes in electrons or energy between the targeted molecules and LMOFs. Persistent porous structures and functional domains of MOFs lead to reversible pre-concentration of targets, enhancing detection accuracy and selectivity. MOFs’ luminous properties and unique shapes provide excellent possibilities for developing revolutionary fluorescent materials for evaluating food quality and safety [89,90]. LMOFs may be created with different reconnaissance moieties to recognize the intended molecules. Conventional solvothermal procedures have been used to create luminous MOFs for hazardous chemical species, volatile organic chemicals, biomolecules, gases, and target conditions (pH, temperature, and moisture) [91].

**Figure 9:**
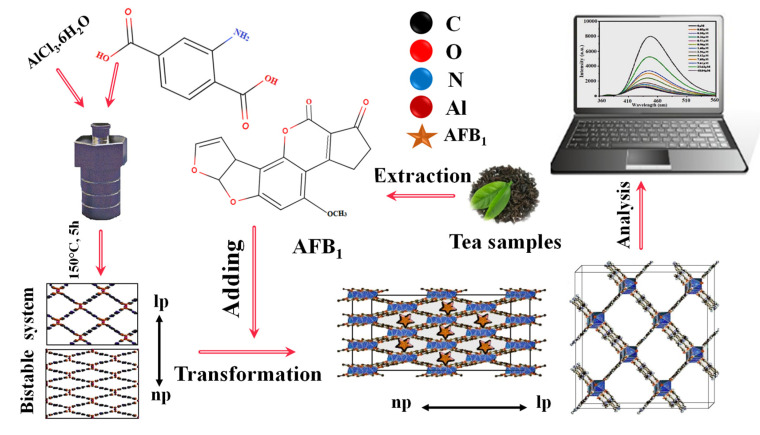
Graphic depiction of fluorescent aflatoxin B1 detecting employing fluorescent metal-organic frameworks. Adapted with permission from the article [96]. 2022, Elsevier.

LMOFs are suitable for the complex food environment, as mycotoxins are found in many foods, and their effects on people and animals are highly variable. Even low doses of mycotoxins can cause cancer, liver disease, and death in humans. Therefore, mycotoxin detection is vital for human health [92]. Due to the flexible structure of LMOFs, multiple fluorescent centers can interact with one another to adjust fluorescence and generate new dual emissions for radiometric sensors. With the development of numerous fluorescent systems, LMOFs may be utilized in various unanticipated applications in the food industry, providing faster response, increased sensitivity, enhanced tolerance to interference, and potential for distant monitoring [93,94]. Diverse food contamination is a significant factor that impacts food security. Owing to their presence in trace amounts and the intricacy of food matrices, it is challenging to separate and identify them reliably. Optical biosensors based on LMOF composites with diverse functions and structures provide a new option for purifying food matrices and enhancing trace targets, thereby paving the way for the emergence of new supersensitive and productive technologies for food safety diagnosis [95]. As a consequence of this, Fuxiang Wang et al. (2022) [96] developed a switching-on fluorescent sensor platform on an ultralow intensity-based Al-MOF for the sensing of AFB1 in various tea samples, including Biluochun tea, Junshan silver needle tea, and Pu’er tea with 0.05–9.61 μM linearity concentration-response range and a 0.01167 mg/L low detection limit (Figure 9). In another study, Ouyang et al. (2022) [97] prepared Co SASCs on ZIF-8@SiO_2_ nanoparticles using a unique in situ etching technique without requiring high temperature. The porous microreactors of Co single-atom site catalysts can accelerate H_2_O_2_ decomposition and create large amounts of superoxide radical anions, increasing the chemiluminescent response of the luminol system. This platform can identify aflatoxin B1 for medication safety with a quantitation range of 1.0 × 10^−5^ to 0.001 mg/L and the 4.4 × 10^–7^ mg/L (3σ) in Glycyrrhiza uralensis and Panax notoginseng. Additionally, Jia et al. (2020) [98] developed a simple fluorescence biosensor based on an aptamer labeled with a fluorophore (TAMRA) and UiO-66-NH_2_. This biosensor can sense AFB1 in milk, rice, and corn samples. The fluorescence emission intensity of the UiO-66-NH_2_/TAMRA aptamer was shown to have a positive connection with that of AFB1 in the concentration range of 0–0.18 mg/L, with a detection limit (LOD) of 0.35 × 10^−3^ mg/L. In addition, by combining Zr-LMOF with a rigid melamine sponge, Li et al. (2021) [99] created a responsive hybrid sponge to collect/remove and simultaneously identify various mycotoxins, including aflatoxin M1, aflatoxin B1, aflatoxin B2, aflatoxin G1, aflatoxin G2, and ochratoxin A, particularly AFB1, in actual samples. The sensor has shown a 0.1 µM to 50 µM linearity range and a 0.0016 mg/L detection limit in peanut products and corn samples. Similarly, Yu and Li (2022) [100] developed a Tb^3+−^ COF that can be configured as a switch-on fluorescence sensor for selective monitoring and sensitivity of ochratoxin A in wheat samples. This was accomplished using the switch-on fluorescence technique. The sensor was developed as a lanthanide-based Dpy/NhBt-COF by anchoring Tb^3+^ onto a 2D imine COF (Dpy/NhBt-COF@Tb^3+^) and demonstrated a low detection limit of 0.0135 M.

Furthermore, in 2021, Tan et al. [101] created a fluorescent signal amplification aptasensor for detecting T-2 mycotoxin in wheat flour and corn. The D-aptamer, tagged with dual/terminal fluorescein amidite (FAM), served both as a sensor-detecting item and a signal indication. MOFs containing N,N′-bis(2-hydroxyethyl) dithiooxamide copper (H_2_dtoaCu) were employed as quenchers. The initial adsorption of the D/aptamer onto the surface of H_2_dtoaCu renders the aptasensor inactive. The D/aptamer undergoes a conformational change upon the addition of T-2 to produce T-2/T-2 aptamer complexes, resulting in the release of the signaling probe from the H2dtoaCu surface. Thus, the intensity of the D/fluorescence aptamer was obtained with a 0.39 × 10^−3^ mg/L lower detection limit and a linearity range of 0.005–0.1 mg/L compared to the single/terminal-FAM/labeled aptamer (S/aptamer). Similarly, Zhao et al. (2021) [102] modified MIL-101 and the surface electric potential of upconversion nanoparticles to construct a FRET-based aptasensor for T-2 toxin sensitivity (UCNPs). Additionally, it combined effective adsorption quenching and MOF capabilities with the excellent spectral features of UCNPs. Through p-p stacking interactions, the UCNPs/aptamer was adsorbed onto the surface of MIL-101, decreasing the fluorescence due to FRET. The T-2 toxin was selectively attached to the UCNPs/aptamer, which led to their migration away from MIL-101 and the restoration of fluorescence emission intensity. In samples of beer and maize meal, this sensor exhibited a detection limit (LOD) of 8.7105 mg/L (S/N = 3) with a linear correlation range of 0.001–0.1 mg/L.

**Figure 10:**
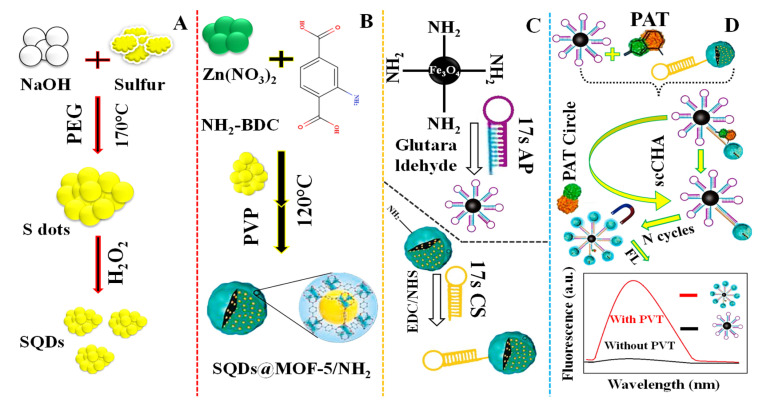
(**A**) Depiction of the SQDs synthesis procedure: (**B**) Diagram of the SQDs@MOF-5-NH_3_ preparation method; (**C**) The DNA hairpin coupling mechanism involving Fe3O4-NH2 and SQDs@MOF-5-NH_2_; (**D**) Demonstration of the fluorescent aptasensor for sensing of patulin (PAT). Adapted with permission from the article [103]. 2022, Elsevier.

For the sensing of patulin (PAT) in apple juice, Yan et al. (2021) [103] provided a fluorescent aptasensor based on sulfur quantum dots encapsulated in a self-cycle-catalyzed hairpin assembly (scCHA) and MOF-5/NH_2_ (SQDs@MOF-5/NH_2_) (Figure 10A). The sensor, related to water-soluble SQDs encapsulation, (SQDs@MOF-5/NH2) produced by “bottle-around-ship” solvothermal approach, works as a fluorescent probe with a 7.53 × 10^−7^ mg/L limit of detection. Figure 10A–C illustrates the SQDs synthesis procedure, the SQDs@MOF-5-NH_3_ preparation method, and the DNA hairpin coupling mechanism involving Fe_3_O_4_-NH_2_ and SQDs@MOF-5-NH_2_, respectively. The fabrication process of the fluorescent aptasensor for the sensing of PAT is shown in Figure 10D.

In another work, Yang et al. (2020) [104] produced a three-dimensional Zn-MOF made from a bipyridyl-tetracarboxylic ligand consisting of four-fold interpenetrated fluorescence to sense 3-nitropropionic acid with a 10 M detection limit and pH-triggered photonic switching (pH = 5.4) in a moldy sugar cane. Table 2 demonstrates recent research based on optical techniques for determining diverse mycotoxin types in food samples.

## 5. Conclusions

Mycotoxins pose significant risks to international public health and agricultural economic growth. Low quantities of mycotoxins in food crops make their detection more challenging. The detection approach that produces useful analytical findings depends on several factors, including sensitivity, detection limit, economic performance, sample-to-result time, and the necessity for sample preparation or extraction. The latest developments in the synthesis of MOFs and NPs have resulted in an increasing number of MOF-based sensors with novel and specialized properties. MOFs are functional materials that exhibit physicochemical properties that are not observed in traditional porous structure materials. MOFs are excellent candidates for applications in food safety investigations because of their structural modularity, post-synthetic form and function, and highly regulated porosity. They also provide significant prospects for sensing and absorption in several domains, including agribusiness, pharmaceuticals, environmental sciences, bioanalytical studies, and food and nutrition security. Several MOFs have been developed, constructed, and implemented in food and nutrition security studies. To improve the robustness and responsiveness of MOFs in intricate samples, post-synthetic alterations have focused on the functionalized signals generated by nanomaterials, such as quantum dots, silver nanoclusters, gold nanoparticles, nanorods, magnetic beads, and the integration of biomolecules. The development of food safety sensors based on MOFs has benefited significantly from this post-synthetic functionalization. Therefore, it is essential to conduct further research to enhance MOF-based sensor designs for food safety assessments that have been disclosed.

## 6. Future Outlooks

Many studies have found that MOFs are effective quality-assurance sensors. However, opposite conclusions have also been drawn on a few occasions. This may be due to variations in experimental settings and surface functional group interactions. In this regard, MOF stability, specificity, and selectivity are the most important characteristics necessary for an analytical sensor to fulfill food safety requirements. In addition, the food business necessitates the availability of inexpensive, user-friendly analytical sensing technologies at every stage of the food supply, even in distant places, with sufficient detection capability. In this context, practical cooperation across multiple research fields could overcome possible obstacles and develop intriguing MOF sensors for food security and safety assessments. The multiple-scientific integration strategy will result in logical and advantageous MOF-based detection techniques that are easy to use at the point of care, affordable, mobile, rigorous, and sensitive, with the ability to detect multiple analytes and remove and absorb contaminants from food without tainting it.

## Figures and Tables

**Table 2:** Recent advances in optical-based sensing platforms to detect various mycotoxins in numerous food samples.

MOF Type	Target Mycotoxins	Fluorescence Response	Detection Limit (LOD)	Linear Range	Recovery (%)	Real Sample	Reference
Zr-MOF	Ochratoxin A (OTA)	Turn-on	2.4 × 10^−8^ mg·L^−1^	1.0 × 10^7^−0.14 × 10^−3^ mg·L^−1^	-	Corn	[67]
NH2-MIL-53	Aflatoxin B1 (AFB1)	Turn-on	0.01167 mg·L^−1^	0.05–9.61 μM	80.99 ± 0.67 to 115.29 ± 3.85	Biluochun tea Junshan silver needle tea Pu’er tea	[96]
Co/NCNT/ZIF-8@ZIF-67	Ochratoxin A (OTA)	Turn-on	1.7 × 10^−7^ mg·L^−1^	1.0 × 10^−6^–0.01 mg·L^−1^	95.0−103.8	Corn and Millet	[70]
Co SASCs derived from ZIF-8@SiO_2_	Aflatoxin B1 (AFB1)	Turn-off	4.4 × 10^−7^ mg·L^−1^	1.0 × 10^−5^−0.001 mg·L^−1^	G·uralensis 88.4−95.4 P·notoginseng 94.4−111.9	Glycyrrhiza uralensis and Panax notoginseng	[97]
MOFs (H2dtoaCu) MOF (H2 dtoa = dithiooxamide anion)	T-2 mycotoxin	Turn-on	0.00039 mg·L^−1^	0.1–0.5 mg·L^−1^	Corn flour 98.02 ± 4.76 to 100.11 ± 3.32 Wheat flour 99.05 ± 1.33 to 00.40 ± 2.82	Corn flour and Wheat flour	[101]
SQDs@MOF-5-NH_2_	Patulin (PAT)	Turn-on	7.53 × 10^−7^ mg·L^−1^	1.0 × 10^−6^–0.1 mg·L^−1^	89.03–107.67	Apple juice sample	[103]
Dpy-NhBtCOF@Tb^3+^	Ochratoxin A (OTA)	Turn-on	0.0135 μM	0–10 μM	97.3–98.6	Wheat	[100]
TAMRA aptamer/UiO-66-NH_2_	Aflatoxin B1 (AFB1)	Turn-on	0.35 × 10^−3^ mg·L^−1^	0–0.18 mg·L^−1^	Milk 103.10–10414 Corn 96.38–97.36 Rice 90.42–94.21	Milk Corn Rice	[98]
Zr-LMOF	Aflatoxin B1 (AFB1)	Turn-off	0.01997 mg·L^−1^	0.0312–15.6 mg·L^−1^	-	Water	[99]
Zr-LMOF/M.F.	Aflatoxin B1 (AFB1)	Turn-off	0.0016 mg·L^−1^	0.0234–7.8 mg·L^−1^	-	Water	[99]
Zr-CAU-24	Aflatoxin B1 (AFB1)	Turn-off	0.01997 mg·L^−1^	75–25,000 μM	91–108	Walnut beverage Almond beverage	[105]
[Zn2(bpdc)2(tppe)]LMOF-241	Aflatoxin B1 (AFB1) Aflatoxin B2 (AFB2) OchratoxinA	Turn-off	0.046 mg·L^−1^	-	-	-	[106]
NH2–UiO–66/Cy3–aptamer	T-2 mycotoxin	Turn-on	0·239 × 10^−3^ mg·L^−1^	0.5 × 10^−3^–0.1 mg·L^−1^	Milk 89.86–108.99 Beer 99.17–111.51	Milk and Beer	[107]
DNA-templated AgNCs/MOFderived/Fe_3_O_4_/carbonoctahedra	Zearalenone [44]	Turn-on	2 × 10^−6^ mg·L^−1^	1.0 × 10^−5^–0.25 mg·L^−1^	Maize 97.3–102.9 Wheat 96.0–101	Maize and Wheat	[108]
Dye-functionalized MOF (FITC@1)0(Cd(NO_3_)_2_·6H_2_O/DMF/C_2_H_5_OH) = 1	3-nitropropionic acid (3-NPA)	Turn-off	135,000 μM	-	-	Moldy sugarcane	[109]
MPC–NGQDs-Ap	Ochratoxin A (OTA)	Turn-on	0.00405 mg·L^−1^	0.01–5 mg·L^−1^	-	Wheat and Corn	[110]
MPC–NGQDs-Ap/exonuclease I (ExoI)	Ochratoxin A (OTA)	Turn-on	0.00228 mg·L^−1^	0.01–5 mg·L^−1^	wheat 86.66–99.14 Corn 96.06–109.9	Wheat and Corn	[110]
MIL53-SiO_2_@Fe_3_O_4_	Aflatoxin B1 (AFB1)	Turn-on	0.0005 mg·L^−1^	0.0005–0.15 mg·L^−1^	70.7–96.5	Linden Chamomile Purple a Purple Easternholly Cinnamon Clove Sage leave Lemon Ginger	[111]

## Data Availability

No new data were created or analyzed in this study. Data sharing is not applicable to this article.
